# Patterns of multimorbidity among low-income adults who smoke with implications for tailored interventions: a cluster analysis using a Mixture of Bernoulli model

**DOI:** 10.3389/fmed.2026.1735343

**Published:** 2026-03-31

**Authors:** Monique T. Cano, Michael R. Lindstrom, Oscar F. Rojas Perez, Ricardo F. Muñoz

**Affiliations:** 1 Department of Psychiatry, Yale University School of Medicine, New Haven, CT, United States; 2 Institute for International Internet Interventions for Health (i4Health), Palo Alto University, Palo Alto, CA, United States; 3 Department of Psychiatry and Behavioral Sciences, University of California, San Francisco, San Francisco, CA, United States; 4 School of Mathematical and Statistical Sciences, The University of Texas Rio Grande Valley, Edinburg, TX, United States

**Keywords:** cluster of co-morbidity, low-income adults, multimorbidity, primary care, smoking, tobacco-use, underserved populations

## Abstract

**Introduction:**

Smoking cigarettes remains a leading modifiable risk factor for preventable health conditions. In the United States, the health burden of smoking disproportionately impacts low-income individuals. Multimorbidity is common in this group, complicating treatment and worsening outcomes. Identifying multimorbidity clusters can support targeted, individualized interventions. This study aimed to identify multimorbidity clusters among individuals who smoke and experience economic hardship and provide clinical recommendations to enhance health outcomes.

**Method:**

Individuals who smoke and experience economic hardship (*N* = 60) were recruited from the San Francisco Health Network (SFHN) and were assessed for physical and mental conditions. Cluster analysis was conducted using a Mixture of Bernoulli (MoB) model to identify subgroups of participants based on co-occurring physical and mental health conditions. Relative risks (RRs) were calculated to compare the likelihood of each condition across clusters, and 95% confidence intervals were used to assess statistical significance.

**Results:**

Cluster analysis identified three groups: *Physical Multimorbidity*, *Mental-Physical Multimorbidity*, and *Lower Health Burden*. Gender was significantly associated with cluster membership: males were more likely to be in the *Physical Multimorbidity* cluster, and females were more likely to be in the *Mental-Physical Multimorbidity* cluster (*p* < 0.01). Findings should be interpreted cautiously given the small sample size and exploratory nature of the cluster analysis.

**Conclusion:**

Individuals who smoke and experience economic hardship exhibited three multimorbidity clusters—*Physical Multimorbidity*, *Mental-Physical Multimorbidity*, and *Lower Health Burden*—indicating both overlapping and distinct patterns of chronic health conditions. Chronic pain was common across the more complex clusters, whereas depression and anxiety characterized the *Mental-Physical Multimorbidity* cluster. These findings highlight the need for tailored and person-centered smoking cessation strategies that address both shared and unique physical and mental health challenges in this high-risk population.

## 1 Introduction

The health consequences of smoking have been clearly outlined for over six decades (1). However, smoking cigarettes continues to be a significant modifiable behavioral risk factor and a leading cause of preventable morbidity and mortality (2), with some populations continuing to be disproportionately affected by tobacco use (3). In the United States, the disease burden associated with smoking has become more concentrated among underserved low-income populations (4). People who smoke and experience economic hardship often face multiple barriers, including limited access to health insurance and healthcare, residence in neighborhoods with high tobacco retail density, and lower formal education (3, 5–7). These structural and social disadvantages increase their risk of developing smoking-related non-communicable diseases (NCDs) (8).

Existing studies on the relationship between smoking and incidence of smoking-related diseases are prolific (4, 9, 10). Prior research indicates that individuals who smoke and experience economic hardship bear a greater health burden from tobacco use and have more physical and mental health conditions than the general population (11–13). Multimorbidity—the co-occurrence of two or more long-term physical and/or mental health conditions—(14, 15) is associated with tobacco-related health disparities (16, 17), and smoking is a well-documented risk factor for multimorbidity (18, 19). These co-occurring conditions can be understood within the biopsychosocial model, which emphasizes that biological, psychological, and social factors are interconnected in shaping health outcomes (20). Framing multimorbidity through this perspective is particularly relevant for populations experiencing economic disadvantage, as it highlights how structural, social, psychological, and biological influences collectively shape health outcomes (21, 22). This whole-person perspective also underscores the importance of addressing modifiable behaviors, such as smoking, which can exacerbate multimorbidity and complicate chronic disease management (23).

Smoking can exacerbate the burden of multimorbidity as it also interferes with the management of existing chronic conditions, including treatment adherence and chronic disease self-management (4, 24, 25). As a result, smoking represents a critical intervention target among individuals with multimorbidity; sustained smoking cessation may improve adherence to treatment regimens, reduce health complications, lower healthcare costs, and improve quality of life (26). Identifying patterns of multimorbidity is an important step toward developing targeted interventions for individuals who smoke and manage multiple chronic conditions. This knowledge can guide clinical conceptualization, inform tailored treatment planning, and support more precise clinical decision-making. Tailoring treatment reinforces person-centered care practices (27) and can improve both prevention and treatment outcomes (28–30). For individuals who smoke and experience economic hardship, such approaches may help reduce health inequities by improving preventive services and strengthening long-term engagement in care (31, 32).

Despite research documenting the health burden of smoking and the prevalence of multimorbidity (10, 33, 34), existing studies on smoking and disease have largely examined smoking-related diseases individually (35–39). The prevalence of multimorbidity has been documented among socioeconomically disadvantaged populations and subgroups (40), and associations between sociodemographic and behavioral factors (including smoking) and multimorbidity have been examined using cross-sectional data (41, 42). However, relatively little is known about how chronic conditions co-occur specifically among individuals who smoke and experience economic hardship. Accordingly, this study aimed to identify multimorbidity clusters among individuals who smoke and experience economic hardship to inform clinically actionable recommendations to enhance health outcomes in this high-risk population.

## 2 Materials and methods

### 2.1 Participants and procedures

The current study is based on secondary analyses from a larger outcome study designed initially to better understand factors—depression, anxiety, nicotine dependence, and smoking abstinence self-efficacy—that influence smoking behavior among low-income safety-net patients who smoke (43). Participants were recruited from primary care clinics within the San Francisco Health Network (SFHN). Additional recruitment was conducted at community-based organizations serving adults experiencing economic hardship. Recruitment materials were displayed in clinic waiting areas and other approved locations. Staff at different recruitment sites were provided with flyers to inform potential participants about the study. Eligible individuals were English-speaking adults (≥18 years) who currently smoked cigarettes, had considered or intended to quit smoking in the next 30 days, and who met the low-income threshold for the San Francisco Bay Area (44). All study procedures were approved by the Institutional Review Boards of the University of California, San Francisco, and Palo Alto University.

### 2.2 Measures

#### 2.2.1 Demographic questionnaire

Demographic variables collected included age, gender, race/ethnicity, marital and employment status, total household income, and years of education.

#### 2.2.2 Physical health conditions

Chronic physical conditions were assessed using a self-report questionnaire adapted from the physical health module developed by Atwoli et al. (45). Participants indicated the presence or absence of 22 chronic illnesses experienced within the past year (condition presence was Boolean, false for absence and true for presence, regardless of severity). Conditions included high blood pressure, high cholesterol, heart attack, stroke, heart disease, asthma, seasonal allergies, other lung disease, diabetes, thyroid condition, osteoporosis, acid reflux, ulcer, obesity, arthritis, chronic pain, frequent headaches, neurological disease, epilepsy, cancer, kidney disease, and an option to specify additional conditions not listed.

#### 2.2.3 Mental health conditions

Mental health conditions were assessed using the Generalized Anxiety Disorder—7 (GAD-7) (46) and the Patient Health Questionnaire (PHQ-9) (47). Based on prior research, participants who scored ≥ 8 on the GAD-7 or ≥ 10 on the PHQ-9 were considered to screen positive for generalized anxiety disorder or major depressive episode, respectively (43).

### 2.3 Analyses

#### 2.3.1 Filtering

Participants were removed from the dataset if their physical and mental health diagnosis data were incomplete.

#### 2.3.2 Mixture of Bernoulli model

A Mixture of Bernoulli (MoB) (48) is a statistical method that models a population with Boolean covariates as a mixture of multiple clusters. In some groups it may also be called Latent Class Analysis (49). Individuals each belong to one latent cluster, and their cluster membership is based on a fixed probability distribution. Each cluster has its own probabilities for a given covariate. The overall model is then able to describe the composition of the population and the covariate probabilities for each cluster. Using Maximum Likelihood and the Akaike Information Criterion (AIC) (50), a number of clusters and corresponding probabilities can be found to parsimoniously describe the dataset, i.e., finding an accurate representation while also minimizing the number of parameters. In this study, the population consists of individuals who smoke and experience economic hardship, and the covariates are the presence/absence of different health conditions, such as high blood pressure. The model amounts to identifying: (1) the number of distinct clusters, (2) the probability an individual selected at random belongs to any given cluster, and (3) the probability of each comorbidity within each cluster. More intuitively, we can think of the population as composed of multiple groups, each with its own risk profile (e.g., the probability of high blood pressure), and every member of the population belongs to only one group.

A MoB model is appropriate for our dataset because the health condition data are Boolean and not continuous, so other popular clustering methods like K-means (51), which are distance based, are not suitable. Since our sample size is small and the model is complex, AIC was used as a model selection criterion to avoid underfitting (52). Monte Carlo validations we present provide evidence that this approach neither underfits nor overfits.

#### 2.3.3 Cluster analysis

Cluster analysis was conducted using a Mixture of Bernoulli (MoB) model (53), splitting the population into Clusters 1, 2, and 3. Through a hard clustering of participants from the MoB results, Relative Risks (RRs) were calculated (54) to estimate the probability of having a diagnosis of each physical and mental condition *relative to the probability of having that diagnosis for Cluster 3 members*. When the 95% confidence interval for a RR (54) did not include 1, the condition is identified as having a relative risk that is different from Cluster 3 in a statistically significant manner. Cluster analyses were implemented using the numpy Python library (55). After cluster membership was established, exploratory analyses compared demographic characteristics between clusters using one-way ANOVAs for continuous variables and chi-square tests of independence for categorical variables. To control for Type I error during multiple comparisons, Bonferroni adjustments (56) were applied to all post-hoc pairwise tests. Furthermore, adjusted standardized residuals were examined for chi-square tests to identify specific cell differences, with values exceeding ± 1.96 considered significant at the.05 level. Effect sizes, including Cramér’s V for categorical data and partial eta squared for continuous data, were reported to facilitate interpretation beyond p-values. Exploratory analyses were conducted using IBM SPSS Statistics for Macintosh Version 31 (IBM Corp., Armonk, NY, United States).

Our choice of hard clustering (i.e., assignment based on the maximum posterior probability) is supported by the high cluster assignment certainty as quantified with Average Posterior Probability (AvePP). AvePP is defined as the average posterior probability of group membership among those classified as belonging to that group, with values above 0.7 being indicative of well-separated (probabilistically distinct) classes (57). The respective Average Posterior Probabilities for clusters 1-3 are 0.963, 0.996, and 0.981, respectively.

#### 2.3.4 Model specification and validation

Each MoB fit was done with 500 restarts, with a stopping condition of either having a relative change of less than 10–5 in log likelihood or exceeding 500 Expectation Maximization steps.

Validation of mixture models is often handled via Monte Carlo Simulation (58). For *k* = 1, 2, 3, and 4 clusters, we fit for model parameters on the true dataset using Maximum Likelihood, generating four sets of parameters. For each set of parameters, we ran 1,000 trials where a new, synthetic dataset for *N* = 60 fake patients was generated and AIC was used to identify the optimal number of clusters (within the range of 1–5) for that dataset. We then compared the true number of clusters used to generate the data with the optimal number of clusters identified through AIC as seen in Table 1. Such approaches have been used to assess statistical power in latent models (59).

**TABLE 1:** Study of number of clusters selected by AIC.

	True number of clusters			
Number of clusters selected	1	2	3	4
1	97%	0%	0%	0%
2	3%	97.4%	15.9%	13.1%
3	0%	2.6%	84.1%	52.7%
4	0%	0%	0%	34.2%
5	0%	0%	0%	0%

Study of the number of clusters selected by AIC when the true number of clusters generating the data range from 1–4. Data generation stems from fitting MoB to the dataset and then generating data with the corresponding number of clusters.

When the number of clusters used to generate the data was 1 and 2, the AIC trials recovered the correct number of clusters with 97 and 97.4% accuracy, respectively, indicating that AIC does not spuriously over-extract classes under these conditions. When 3 clusters were used to generate the data, the AIC trials recovered 3 clusters in 84.1% of the trials, demonstrating a high recovery rate of a three-class structure under the observed parameter configuration. On the other hand, when 4 clusters were used to generate data, 4 clusters were only selected 34.2% of the time, whereas 3 clusters were selected 52.7% of the time. This suggests that a four-class structure, with our estimated parameters, does not yield clusters that are sufficiently distinct for the AIC to select 4 clusters. Such an outcome is consistent with the fourth class being generated by the splitting of a pre-existing class, rather than being an additional, stable, latent group (results not included with 4 clusters create a group with membership probability less than 2%—effectively a single individual forms a new cluster).

Using Parametric Bootstrapping (60), we calculated the 95% confidence intervals for all model parameters with 3 clusters. Results are in Table 2. Class ordering could vary between trials, so from our best fitted MoB parameters Θ, on each of the 1,000 trials, the fitted optimal parameters were permuted so as to minimize the sum of squared differences of vector components between the trial fit Θ* and Θ.

**TABLE 2:** Parametric Bootstrap confidence intervals.

Parameter	Low 1	Up 1	Low 2	Up 2	Low 3	Up 3
Fraction	0.05	0.258	0.099	0.3	0.526	0.786
Heart disease	0	0.74	0	0.286	0	0.028
High blood pressure	0	0.854	0.2	0.859	0.009	0.224
Stroke	0	0.406	0	0	0	0.025
High cholesterol	0	0.091	0	0.581	0	0.172
Heart attack	0	0.397	0	0.333	0	0.024
Seasonal allergies	0.138	1	0.221	0.858	0.085	0.366
Asthma	0	0.796	0	0.483	0.052	0.31
Other lung disease	0	0.074	0	0.418	0	0.077
Acid reflux	0	0.865	0.133	0.786	0	0.145
Obesity	0	0.577	0.144	0.797	0	0.175
Ulcer	0	0.745	0	0	0	0.028
Diabetes	0	0	0	0.467	0	0.125
Thyroid	0	0	0	0.307	0	0
Osteoporosis	0	0	0	0	0	0
Arthritis	0	0.749	0.331	1	0	0.096
Chronic pain	0.367	1	0.497	1	0	0.124
Frequent headaches	0	0.871	0	0.076	0	0.096
Kidney disease	0	0.4	0	0.3	0	0.026
Neurobiological condition	0	0.437	0	0.332	0	0.025
Epilepsy	0	0.4	0	0	0	0.087
Cancer	0	0.4	0	0	0	0.024
Any other illness	0	0.66	0	0.313	0	0.079
Anxiety	0	0.49	0.857	1	0.13	0.41
Depression	0	0.166	0.762	1	0.032	0.263

The 95% confidence intervals for model parameters by cluster, where for example, “Low X” is the 2.5-percentile and “Up X” is the 97.5-percentile of cluster X. “Fraction” is the portion of the population comprised of the given group. The other physical/mental health conditions indicate the probability of having that condition within a given group.

## 3 Results

### 3.1 Participant characteristics

The participant sample consisted of 60 individuals who smoke and experience economic hardship, with an average age of 44.95 ( ± 10.54) years. The mean years of educational attainment was 12.05 ± 2.82 years. Most participants identified as cisgender males (*n* = 39; 65.0%) and one third identified as cisgender females (*n* = 20; 33.3%), with one participant identifying as transgender. Participants identified at Non-Hispanic White (*n* = 20; 33.3%), African American/Black (*n* = 19; 31.7%), Native American/Alaskan Native (*n* = 4; 6.7%), Native Hawaiian/Pacific Islander (*n* = 2; 3.3%), Mestizo (*n* = 2; 3.3%), Biracial (*n* = 11; 18.3%), and an unknown or unreported racial identity (*n* = 1; 1.7%). A quarter of the sample (*n* = 15; 25%) identified as Hispanic/Latinx. Two thirds of participants indicated that were single or had never been married (*n* = 36; 60%), followed by married or in a domestic partnership (*n* = 10; 16.7%), separated (*n* = 4; 6.7%), divorced (*n* = 7; 11.7%), or widowed (*n* = 3; 5.0%). The majority of participants were unemployed (*n* = 43%; 71/7%) and had a total household income of below $20,000 annually (*n* = 48; 80.0%). A summary of the sample characteristics is provided in Table 3.

**TABLE 3:** Characteristics of the sample.

Demographics	Total *N* = 60
Gender n (%)	
Male	39 (65.0)
Female	20 (33.3)
Transgender	1 (1.7)
Age (mean, SD)	44.95 (10.54)
Education (mean, SD)	12.05 (2.82)
Race n (%)	
Non-Hispanic White	20 (33.3)
African American/Black	19 (31.7)
Biracial	11 (18.3)
Native American/Alaskan Native	4 (6.7)
Mestizo	2 (3.3)
Native Hawaiian/Pacific Islander	2 (3.3)
Asian	1 (1.7)
Unknown/Not reported	1 (1.7)
Ethnicity n (%)	
Not Latinx/Hispanic	35 (58.3)
Latinx/Hispanic	15 (25.0)
Unknown/Not reported	10 (16.7)
Marital status n (%)	
Single (never married)	36 (60.0)
Married, or in a domestic partnership	10 (16.7)
Separated	4 (6.7)
Divorced	7 (11.7)
Widowed	3 (5.0)
Employment status n (%)	
Full-time	6 (10.0)
Part-time	3 (5.0)
Retired	2 (3.3)
Student	6 (10.0)
Unemployed	43 (71.7)
Household Income n (%)	
< $20,000	48 (80.0)
$20,000–$34,999	4 (6.7)
$35,000–$49,999	3 (5.0)
$50,000–$74,999	2 (3.3)
$75,000–$99,000	2 (3.3)
Unknown/Not reported	1 (1.7)

### 3.2 Mixture of Bernoulli analysis

**FIGURE 1:**
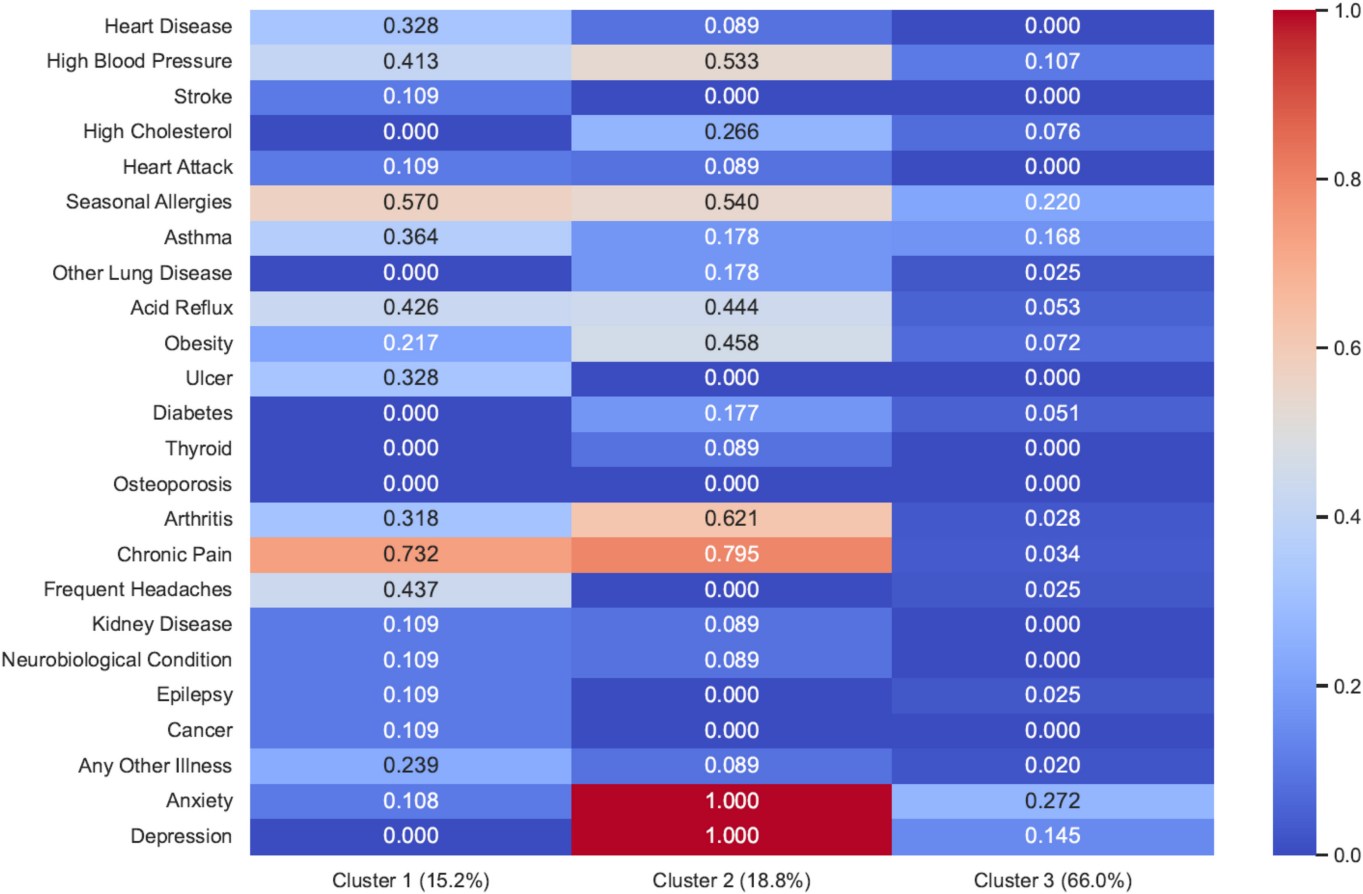
Patterns of multimorbidity across three identified clusters. Patterns of physical and mental health conditions across three clusters (Group 1—*Physical Multimorbidity*, Group 2—*Mental-Physical Multimorbidity*, Group 3—*Lower Health Burden*) identified by the MoB model.

Based on participants’ self-reported physical and mental health conditions, the MoB Model allowed us to place each patient into one of three clusters, (1) Physical Multimorbidity, (2) Mental-Physical Multimorbidity, and (3) Lower Health Burden (Figure 1)—these clusters are labeled as such based on our interpretation of the RRs. The choice of 3 clusters stems from the AIC scores for the number of clusters chosen (Table 4), where the relative support for 3 clusters is about 70% (61). Using the 3-cluster model, we then calculated the RRs for the different disorders for patients in the *Physical Multimorbidity Cluster* (Cluster 1) and the *Mental-Physical Multimorbidity Cluster* (Cluster 2), relative to the *Lower Health Burden Cluster* (Cluster 3), by performing a hard clustering for each patient (yielding group sizes of 9, 11, and 40, respectively). See Table 5 for the RRs and 95% confidence intervals in parentheses. Disorders for which the 95% confidence window suggests that a group has a higher RR for a disorder relative to the *Lower Health Burden* cluster are marked with an asterisk (those with lower RR were also considered, but none were found).

**TABLE 4:** AIC cluster support.

Number of clusters	AIC	ΔAIC	Relative support
1	950.9	46.1	7 × 10^–11^
2	906.7	1.9	0.3
3	904.8	0	0.7
4	914.5	9.7	0.006
5	926.2	21.4	2 × 10^–5^

Analysis of support for different numbers of clusters. The AIC is shown for each number of clusters considered with ΔAIC being the AIC relative to the minimum AIC score. The relative support is shown rounded to one significant figure showing 3 clusters as the optimal choice.

**TABLE 5:** Relative risks of Groups 1 and 2 relative to Group 3 for different conditions.

Health condition	Group 1 RR (95% CI)	Group 2 RR (95% CI)
Heart disease	28.7 (1.6–512.0)*	10.3 (0.4–235.7)
High blood pressure	4.4 (1.4–14.5)*	5.5 (1.9–16.0)*
Stroke	12.3 (0.5–280.0)	Not computable
High cholesterol	0.6 (0.0–10.4)	3.6 (0.8–15.6)
Heart attack	12.3 (0.5–280.0)	10.3 (0.4–235.7)
Seasonal allergies	2.5 (1.1–5.6)*	2.4 (1.1–5.3)*
Asthma	1.9 (0.6–6.0)	1.0 (0.3–4.3)
Other lung disease	1.4 (0.1–31.1)	7.3 (0.7–73.0)
Acid reflux	8.9 (1.9–41.3)*	9.1 (2.0–40.7)*
Obesity	3.0 (0.6–15.2)	6.1 (1.7–21.5)*
Ulcer	28.7 (1.6–512.0)*	Not computable
Diabetes	0.8 (0.0–15.8)	3.6 (0.6–23.0)
Thyroid	Not computable	10.3 (0.4–235.7)
Osteoporosis	Not computable	Not computable
Arthritis	13.3 (1.6–113.8)*	25.5 (3.5–185.5)*
Chronic pain	31.1 (4.4–222.3)*	32.7 (4.6–231.3)*
Frequent headaches	17.8 (2.2–140.7)*	1.1 (0.0–26.2)
Kidney disease	12.3 (0.5–280.0)	10.3 (0.4–235.7)
Neurobiological condition	12.3 (0.5–280.0)	10.3 (0.4–235.7)
Epilepsy	4.4 (0.3–64.6)	1.1 (0.0–26.2)
Cancer	12.3 (0.5–280.0)	Not computable
Any other illness	8.9 (0.9–87.7)	3.6 (0.2–53.6)
Anxiety	0.4 (0.1–2.7)	3.6 (2.2–6.0)*
Depression	0.3 (0.0–5.1)	6.7 (3.2–13.9)*

Disorders for which the 95% confidence window suggests that a group has a higher or lower RR relative to Group 3 are marked with an asterisk. In cases when two groups being compared both have zero patients with the condition, RR cannot be computed, e.g., both Group 2 and Group 3 have zero patients with Stroke.

Compared with the *Lower Health Burden Cluster*, the *Physical Multimorbidity Cluster* had higher proportions of participants with Heart Disease (RR = 28.7; 95% CI [1.6–512.0]), High Blood Pressure (RR = 4.4; 95% CI [1.4–14.5]), Seasonal Allergies (RR = 2.5; 95% CI [1.1–5.6]), Acid Reflux (RR = 8.9; 95% CI [1.9–41.3]), Ulcer (RR = 28.7; 95% CI [1.6–512.0]), Arthritis (RR = 13.3; 95% CI [1.6–113.8]), Chronic Pain (RR = 31.1; 95% CI [4.4–222.3]), and Frequent Headaches (RR = 17.8; 95% CI [2.2–140.7]).

In addition, when compared with other clusters, the *Mental-Physical Multimorbidity* cluster had higher proportions of participants with High Blood Pressure (RR = 5.5; 95% CI [1.9–16.0]), Seasonal Allergies (RR = 2.4; 95% CI [1.1–5.3]), Acid Reflux (RR = 9.1; 95% CI [2.0–40.7]), Obesity (RR = 6.1; 95% CI [1.7–21.5]), Arthritis (RR = 25.5; RR [3.5–185.5]), Chronic Pain (RR = 32.7, 95% CI [4.6–231.3]), and Depression (RR = 6.7, 95% CI [3.2–13.9]).

The *Lower Health Burden* cluster included different chronic clinical conditions with prevalences ranging from 0 to 27.2%. The health conditions with the highest prevalence rates included Anxiety (27.2%), Seasonal Allergies (21.9%), Asthma (16.8%), Depression (14.5%), and High Blood Pressure (10.7%).

As a final set of remarks on the clustering, we observe that the clusters were of unequal size (9, 11, and 40), which has implications for interpretation. First, relative risk estimates for one of the smaller clusters (relative to the large cluster) may appear large because just one or two patients with a given condition can substantially impact the estimated risk in small groups. Second, due to the small cluster sizes and sometimes sparse contingency tables, several of the confidence intervals are wide, reflecting low statistical precision for some RR estimates. Multiplicity corrections, such as Bonferroni, were not applied in the RR estimates because these analyses are exploratory, aiming to describe the broad patterns of disease prevalence across clusters; given the small sample size and sparse event counts, such corrections could substantially increase Type II error. Accordingly, caution should be used in interpreting the clusters and the analysis should be viewed as hypothesis-generating, rather than providing confirmation of the clusters. Finally, while some of the clusters are small, the Monte Carlo recovery analysis suggests the three clusters are reproducible structures in the observed data.

We conducted a sensitivity analysis by estimating a two-cluster solution, which was the only alternative model with non-negligible support based on AIC criteria (Table 4). This solution consolidated the Physical Multimorbidity and Mental–Physical Multimorbidity clusters and retained the Lower Health Burden cluster (Supplementary Figure S1).

### 3.3 Exploratory analyses

One-way ANOVAs and chi-square tests of independence were conducted to examine differences between clusters on the following demographic variables: Age, years of education, gender, sexual orientation, marital status, employment status, and total household income. To control for Type I error across all post-hoc comparisons, a Bonferroni adjustment was applied. A chi-square test of independence indicated a significant association between self-identified gender and cluster membership, χ^2^(2, *N* = 60) = 15.29, *p* = 0.004, representing a large effect size (Cramér’s *V* = 0.357). Post-hoc pairwise comparisons and adjusted standardized residuals revealed that females were significantly more likely than males to belong to the *Mental-Physical Multimorbidity* cluster (residual = 3.1, *p* < 0.05), while males were more likely than females to be represented in the *Lower Health Burden* cluster (residual = 2.3, *p* < 0.05).

No significant differences were found for age (*p* = 0.112, η*_*p*_*^2^ = 0.074) or years of education (*p* = 0.599, η*_*p*_*^2^ = 0.018). Notably, the medium effect size observed for age suggests that the study may have been underpowered to detect significant differences for this variable despite a moderate underlying association.

## 4 Discussion

### 4.1 Multimorbidity cluster profiles

Building on the recognized need to better understand multimorbidity patterns—especially among priority populations—this exploratory study identified multimorbidity clusters among individuals who smoke and experience economic hardship. To the best of our knowledge, few studies have investigated multimorbidity patterns among groups often underrepresented in research. Results indicated that there were three clusters: (1) *Physical Multimorbidity*, (2) *Mental-Physical Multimorbidity*, and (3) *Lower Health Burden*. Cluster memberships reflect distinct clinical presentations, each shaped by varying combinations of multimorbidity. Recognizing these unique patterns provides a meaningful starting point for guiding tailored and contextually relevant smoking cessation approaches, while also highlighting the importance of personalized care.

While the *Physical Multimorbidity* cluster and the *Mental-Physical Multimorbidity* cluster exhibited some overlaps, distinct patterns emerged in their health profiles. The *Physical Multimorbidity* cluster demonstrated a higher tendency for physical and pain-related conditions with minimal to no mental health concerns. In contrast, the *Mental-Physical Multimorbidity* cluster was characterized by universal depression and anxiety in addition to multiple chronic physical conditions. The differences between the two clusters became clearer through exploratory analyses, which added nuance by examining whether demographic factors influenced the observed clustering patterns.

Exploratory analysis revealed notable gender differences, with men being more likely to belong to the *Lower Health Burden* cluster, characterized by a reduced likelihood of both physical and mental health conditions. In contrast, women were more likely to belong to the *Mental-Physical Multimorbidity* cluster, characterized by universal depression and anxiety along with high rates of chronic pain. This difference is well documented in the literature in that women tend to report and experience higher rates of depression and anxiety (62, 63). Women are more likely to report mild to moderate symptoms of depression and demonstrate a more help-seeking coping style, whereas men are more likely to minimize or underreport emotional distress and tend to delay the acknowledgment of symptoms until they become severe (64). Similarly, a substantial body of research suggests that women are more likely than men to experience and report anxiety, with symptoms that are more severe and longer in duration (65). However, these findings should be interpreted with caution. The sample size for certain gender-by-cluster combinations was small, raising the possibility of cells with *n* < 5 and limited statistical precision in these comparisons. These gender differences may reflect reporting bias, including differences in emotional expression, self-reporting, and measurement, as well as potential clinician bias, as men are less likely to acknowledge distress and/or seek treatment (66). Furthermore, unmeasured socioeconomic and cultural factors could contribute to these patterns, particularly in a sample experiencing economic hardship. Given these methodological considerations and the exploratory nature of the analysis, the results highlight potential trends rather than definitive conclusions.

These findings highlight the importance of incorporating demographic factors—particularly gender—when examining multimorbidity patterns. Moving beyond gender comparisons, an intersectional framework suggests that these patterns are likely shaped by the complex interplay between gender identity and unmeasured social determinants of health (67). This perspective is particularly salient given the sample’s economic hardship, as the results likely reflect a broader constellation of socio-environmental determinants not fully captured by the demographic variables. While no other demographic differences emerged in exploratory analyses, future research with larger cohorts can adopt a multi-dimensional approach to better understand how intersecting social factors shape clustering patterns among underserved populations.

### 4.2 Psychosocial and biological mechanisms contributing to multimorbidity

Notably, both the *Physical Multimorbidity* and *Mental-Physical Multimorbidity* clusters were characterized by higher tendencies for experiencing chronic pain. Individuals with chronic pain who smoke tend to report high levels of pain severity, decreased functional impairment, worse pain interference, and experience higher levels of depression and anxiety symptoms (68). Shared symptomatology and common factors such as negative affect may partially explain the link between these conditions (69). For example, negative affectivity—the subjective experience of negatively-valenced emotions, including but not limited to sadness, anxiety, anger, and fear (70)—has been found to impact both pain intensity and smoking behavior (69). Pain—both physical and emotional—can induce the urge to smoke (71) as nicotine’s psychoactive effects are associated with mood modulation to manage and alleviate internal aversive experiences (72–75). However, the complex relationships between negative affect, chronic pain, and smoking appear to be multidirectional and cyclical, given that smoking is also linked to greater pain severity (68). Pain may increase the urge to smoke, and smoking may temporarily modulate physical and emotional pain; however, this relationship is likely bidirectional.

Chronic stress and anxiety are also strongly linked with pain experiences (76, 77). People who experience economic hardship are at greater risk for experiencing the interconnected effects from stress, anxiety, and pain (78). Empirical evidence suggests that anxiety and conditions that are characterized by pain—whether acute or chronic, such as arthritis, headaches, and gastrointestinal disorders like acid reflux and ulcers—demonstrate a bidirectional relationship; for instance, anxiety may serve as both a precipitating factor and a consequence of ongoing and/or recurrent pain experiences (69, 79, 80). However, somatic symptoms of anxiety—e.g., characterized by abdominal pain, headache, fatigue, dizziness, insomnia, dyspepsia—are among the most frequent reasons for primary care visits (81, 82). It is estimated that over half of patients with anxiety disorders who present to primary care settings may be misdiagnosed, as somatic symptoms can be falsely attributed to underlying medical conditions (82, 83).

The symptom overlap found across clusters may reflect not only psychosocial vulnerabilities but also shared biological mechanisms. Cigarettes contain thousands of chemicals, including over 60 cancer-causing toxins (19), which directly impact the immune system, and long-term cigarette smoking can induce a systemic proinflammatory state (84). Chronic inflammation associated with long-term cigarette smoking is related to chronic diseases such as depression, chronic pain, arthritis, and cardiovascular disease (85–88). Consistent with previous research, chronic inflammation—potentially driven by long-term smoking—may serve as a key biological mechanism underlying the co-occurrence of NCDs such as cardiovascular disease and depression (87, 89), thus maintaining and exacerbating mental-physical multimorbidity. Of note, the biological mechanisms described here are consistent with previous research but were not directly evaluated in the present study.

### 4.3 Mental health needs despite lower multimorbidity burden

While shared biological mechanisms may help explain the clustering of mental-physical multimorbidity among the more complex clusters, the *Lower-Health Burden* cluster exhibited a notably less complex profile, characterized by lower probabilities of both physical and mental health conditions. This group may represent individuals with fewer barriers to smoking cessation. Prioritizing early prevention of physical and mental health conditions may be key to supporting smoking cessation within this group. It is worth noting, however, that over one quarter of individuals in this cluster screened positive for generalized anxiety disorder, and about 14% screened positive for a major depressive episode. This highlights that even among those without complex clinical presentations, targeted attention to mental health remains relevant for supporting smoking cessation.

### 4.4 Clinical implications

Smoking cessation among individuals who smoke, and experience economic hardship is influenced by a range of both individual and structural factors. The specific barriers to quitting vary from person to person, including differences in relapse triggers, withdrawal experiences, and the use of smoking to manage mental health symptoms (90). These challenges are often compounded by socioeconomic disadvantages, which also vary across individuals. In attempting to quit smoking, there are a variety of strategies—such as nicotine replacement therapies, medication, cognitive-behavioral therapy (91), avoiding triggers, peer support, or engaging in alternative behavioral activities (92), which may be effective for some and counterproductive for others (90), underscoring the critical role of tailoring interventions to address the unique needs of each patient.

#### 4.4.1 Individualized cessation strategies

Individualized care for primary care multimorbid patients who smoke can begin by identifying the factors that are most distressing or impairing for the patient, as well as the underlying factors that influence smoking behavior. For example, smoking may function as a strategy for mood regulation and chronic disease self-management (93, 94). Among individuals experiencing both depression and anxiety, greater depressive symptom severity may influence smoking behavior to a larger extent, warranting a stronger emphasis on addressing mood within cessation efforts. Longitudinal evidence suggests that higher depression levels significantly reduces the likelihood of sustained smoking cessation (95), highlighting the need for support before, during, and after quit attempts (96). Interdisciplinary approaches that combine both behavioral smoking cessation interventions and pharmacotherapy are particularly important, as integrated treatments have been shown to improve quit rates when compared to usual care (97–101).

#### 4.4.2 Addressing psychosocial factors and pain in clinical care to support smoking cessation

Beyond cessation outcomes, addressing depression in conjunction with smoking may yield broader health benefits, particularly for individuals represented in the *Mental-Physical Multimorbidity* cluster. Research indicates that sustained smoking cessation can improve chronic illness outcomes, including reduced disease progression from COPD, reduced cardiovascular risk, and reduced mortality from lung cancer (102); however, these benefits are less likely if depression remains unaddressed (103). Additionally, lower-income patients experience higher rates of depression (104), often characterized by greater symptom severity and recurrent depressive episodes, necessitating the need for more intensive, sustained, and targeted treatment. This may involve a coordinated care model that involves referral pathways to ensure continuity of care. For example, primary care providers may refer to behavioral health consultants who then ensure timely connection to mental health services in an effort to sustain comprehensive care rather than fragmented treatment.

Similarly, integrating stress and anxiety management into smoking cessation treatment could enhance engagement and long-term success among individuals whose smoking is primarily driven by anxiety or stress-related symptoms. To improve sustained abstinence rates beyond the primary care clinical encounter, referrals to behavioral health specialists and mental health clinicians are necessary (105). Teaching and practicing relaxation and distress tolerance techniques with patients is an important treatment target; methods such as diaphragmatic breathing, progressive muscle relaxation, and brief cold-water exposure can help regulate stress responses (106, 107). These approaches equip individuals with practical and actionable skills to address stress in real time, helping them navigate smoking urges as they arise (108). Integrating these skills with psychoeducation that emphasizes the short-lived nature of cravings and reinforcing these skills through self-monitoring can further support patients in coping with stress in adaptive ways without using cigarettes (109). In addition to regulating stress and cravings, relaxation and distress tolerance techniques exert secondary treatment effects on pain outcomes and may serve as adjunctive strategies for conditions characterized by pain, such as arthritis (110), further supporting overall symptom management.

Furthermore, given the frequent overlap between anxiety and chronic pain, recognizing these co-occurring symptoms within primary care can serve as a foundation to guide patient-centered education and care planning. For example, providers can maximize the clinical encounter by providing brief clinical advice on the benefits of smoking cessation and its potential to improve long-term pain outcomes across multiple chronic conditions. By incorporating motivational enhancement micro-skills such as ask-offer-ask (86), providers invite patients to engage in reflection, considering the information presented and how it is personally relevant to their symptoms and health goals. Framing smoking cessation as directly relevant to the patient’s presenting symptoms may increase readiness for cessation attempts and engagement in more intensive smoking cessation counseling (105).

#### 4.4.3 Prevention strategies for smoking-related multimorbidity

In contrast to those who experience higher psychological and physical symptom burden, individuals within the *Lower-Health Burden* cluster may require a clinical focus that emphasizes prevention. Given that there are time constraints within the primary care clinical encounter, interventions within this context work best when they are delivered quickly and briefly (111). Early preventative strategies that promote healthy lifestyle behaviors—including regular physical activity, balanced nutrition, adequate sleep, meaningful social relationships, stress management, and reduction in substance use—may support smoking cessation while also reducing risk for future chronic disease (112). Incorporating brief or self-guided approaches, such as online or mobile-based interventions, may further enhance accessibility and engagement among individuals who experience economic hardship and tend to face barriers to care (113, 114). These approaches can also provide a valuable adjunct to smoking cessation, especially for patients who could also benefit from brief, targeted online interventions for mood, stress, or anxiety management (115). Within primary care, clinicians can also emphasize how quitting smoking and adopting healthy lifestyle behaviors can significantly reduce the likelihood of developing NCDs.

### 4.5 Limitations and future directions

The findings of this study are interpreted within the context of limitations. Although the Bernoulli mixture model offers a robust approach to identifying patterns in the data, cluster analysis is an exploratory method, and different analytic approaches may yield different clusters. Second, the relatively small sample size limits generalizability to the broader population of individuals who smoke and experience economic hardship. Third, self-selection bias may have influenced study findings, as participants who chose to enroll could differ from those who did not. Fourth, because the sample consisted of individuals who smoke and experience economic hardship from an urban area in the United States, the findings may not extend to low-income populations in rural settings or in other countries. Fifth, the study relied on self-report measures, and mental and physical health conditions were not confirmed through clinical assessment or formal diagnoses. Sixth, due to the small sample size, there is limited precision in the RRs. Finally, measurement bias may be present in the assessment of anxiety and depression, as cultural and gender factors can influence how symptoms are reported and interpreted. Despite these limitations, the study’s diverse sample of participants, along with the application of a rigorous Mixture of Bernoulli model, provided novel, exploratory insights into multimorbidity patterns among a population disproportionately affected by social determinants of health.

Further research in this topic area is needed. Future studies could assess whether similar or different multimorbidity patterns exist in other low-income populations, including rural communities or international settings, to evaluate generalizability and contextual influences that may impact health profiles. Longitudinal studies may clarify how clinical presentations evolve over time following cessation. Additionally, future studies examining biopsychosocial mechanisms such as chronic inflammation, stress, and neighborhood environment could provide further insight into the biological and psychosocial factors that contribute to cluster membership. By focusing on an underserved population with complex clinical profiles, this work addresses a critical gap in understanding multimorbidity patterns among individuals who smoke and experience economic hardship and lays the foundation for improving prevention and treatment strategies and health outcomes in primary care.

## Data Availability

The datasets presented in this article are not readily available because restrictions apply to the datasets. The datasets for this manuscript are not publicly available due to confidentiality regarding the involvement of a vulnerable population. Requests to access the datasets should be directed to the primary author, MTC, monique.cano@yale.edu.
