# Supplementary material for: Patterns of multimorbidity among low-income adults who smoke with implications for tailored interventions: a cluster analysis using a Mixture of Bernoulli model
Source: Front Med (Lausanne). 2026 Mar 31;13:1735343. doi: 10.3389/fmed.2026.1735343 (PMC13076157; doi:10.3389/fmed.2026.1735343)
Supplement: Supplementary file 1 [file Supplementary_file_1.pdf]

Figure S1

Patterns of Multimorbidity Across Two Clusters

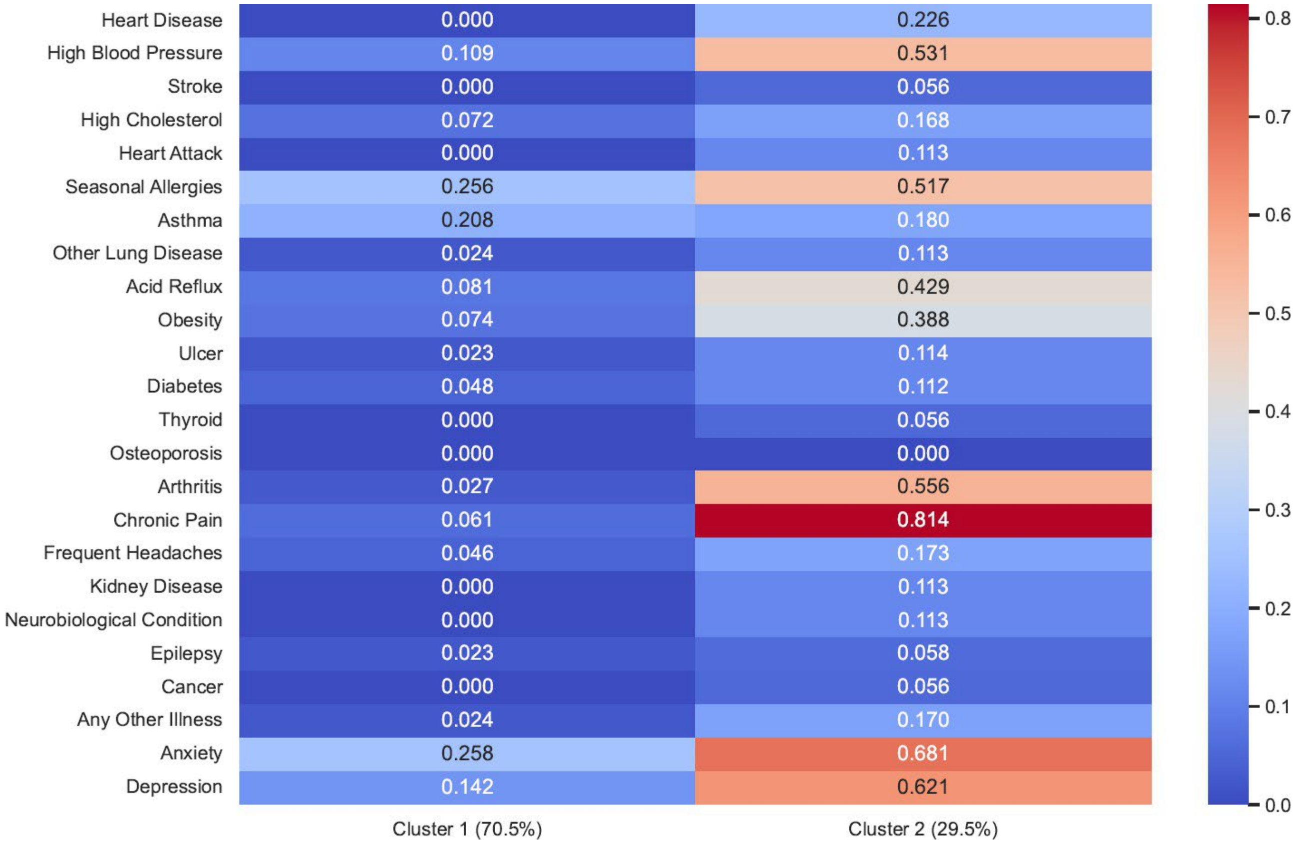

A two-cluster fit for sensitivity analysis: Groups 1 and 2 from the three-cluster fit seem to merge into Group 2 for the two-cluster solution.
